# MC-YOLOv5: A Multi-Class Small Object Detection Algorithm

**DOI:** 10.3390/biomimetics8040342

**Published:** 2023-08-02

**Authors:** Haonan Chen, Haiying Liu, Tao Sun, Haitong Lou, Xuehu Duan, Lingyun Bi, Lida Liu

**Affiliations:** 1 School of Information and Automation Engineering, Qilu University of Technology (Shandong Academy of Sciences), Jinan 250353, China; 10431210436@stu.qlu.edu.cn (H.C.); 10431210427@stu.qlu.edu.cn (H.L.); 10431210431@stu.qlu.edu.cn (X.D.); 10431210560@stu.qlu.edu.cn (L.B.); 2 Shandong Runyi Intelligent Technology Co., Ltd., Jinan 250002, China; zhangjun@raonetech.com

**Keywords:** YOLOv5, multi-class, small objects, shallow network optimization, CB structure

## Abstract

The detection of multi-class small objects poses a significant challenge in the field of computer vision. While the original YOLOv5 algorithm is more suited for detecting full-scale objects, it may not perform optimally for this specific task. To address this issue, we proposed MC-YOLOv5, an algorithm specifically designed for multi-class small object detection. Our approach incorporates three key innovations: (1) the application of an improved CB module during feature extraction to capture edge information that may be less apparent in small objects, thereby enhancing detection precision; (2) the introduction of a new shallow network optimization strategy (SNO) to expand the receptive field of convolutional layers and reduce missed detections in dense small object scenarios; and (3) the utilization of an anchor frame-based decoupled head to expedite training and improve overall efficiency. Extensive evaluations on VisDrone2019, Tinyperson, and RSOD datasets demonstrate the feasibility of MC-YOLOv5 in detecting multi-class small objects. Taking VisDrone2019 dataset as an example, our algorithm outperforms the original YOLOv5L with improvements observed across various metrics: mAP50 increased by 8.2%, mAP50-95 improved by 5.3%, F1 score increased by 7%, inference time accelerated by 1.8 ms, and computational requirements reduced by 35.3%. Similar performance gains were also achieved on other datasets. Overall, our findings validate MC-YOLOv5 as a viable solution for accurate multi-class small object detection.

## 1. Introduction

With the advancement of deep learning technology, numerous object detection algorithms have undergone upgrades and optimization, resulting in their application to various aspects of life after rigorous stability testing. For instance, small object detection algorithms are deployed by road cameras to detect concealed hazards such as nails or stones on the road surface for traffic safety purposes. Ground crew can utilize this technology to identify birds near airports in a timely manner and drive them away to prevent flight accidents. In addition, doctors can use small object detection algorithms through endoscopic instruments to locate abnormal tissues that may be overlooked in patients’ bodies within the medical field. With the advent of RTX series graphics cards, deep learning-based object detection algorithms have observed a significant boost in training speed. Meanwhile, one-stage object detection algorithms such as YOLOv5 [1] are gaining more and more traction due to their advantages over two-stage counterparts such as faster RCNN [2] which are now considered outdated. The performance of deep neural networks on public datasets such as MS COCO [3] has also improved significantly. However, small object detection remains one of the most challenging problems in computer vision due to issues such as sparse visual features, dense distribution or severe occlusion in 2D images. Furthermore, there is a lack of large-scale benchmark datasets for multi-class small object detection, making it difficult for single or multiple types of small object detection algorithms to achieve high precision and performance across different fields.

Researchers have made several advancements in detecting small objects, such as Jing [4] et al.’s algorithm for identifying small traffic objects and Lou [5] et al.’s camera-based detection method. Optimizing algorithms for specific environments offers more benefits than traditional approaches in a single field; however, these improvements have limitations and may not perform well in other scenarios, such as the image of VisDrone2019 [6], the TinyPerson [7,8] dataset for small pedestrians captured by drones or the RSOD [9,10] dataset of high-altitude remote sensing images. When multiple objects are detected, occlusion can occur, leading to a loss of edge features and reduced accuracy. Additionally, variations in light intensity and image angle can cause varying degrees of distortion.

Based on the YOLO algorithm, improvements have been made to the feature extraction, feature fusion, and detection components, resulting in a significant breakthrough. The YOLO series of algorithms have undergone rapid updates and iterations in recent years, placing them at the forefront in terms of speed and precision. However, for practical industrial applications, lightweighting and stability are more important considerations. While [11], YOLOv2 [12], YOLOv3 [13] and YOLOv4 [14] have reached maturity with little room for further improvement, the latest version of YOLOv8 has not yet received widespread certification. As such, MC-YOLOv5—a multi-class small object detection algorithm—was developed as an improved alternative.

The main contributions of the proposed algorithm are as follows:

- The feature extraction process incorporates a novel CB module, which effectively enhances the semantic information of small objects and significantly improves detection precision.
- The SNO was implemented to enhance the receptive field and minimize the rate of missed object detection.
- The decoupled head based on the anchor frame is employed for object classification and localization to enhance reasoning efficiency. Following an extensive evaluation on VisDrone2019, Tinyperson, and RSOD datasets, MC-YOLOv5 demonstrates superior precision and speed compared to the original YOLOv5L.

## 2. Related Work

Current mainstream object detection algorithms can be categorized into one-stage and two-stage algorithms based on whether they use region candidate boxes. While the two-stage algorithm has high detection accuracy, its slow speed makes it unsuitable for real-time scenarios. The one-stage algorithm, pioneered by YOLO algorithm, has achieved remarkable results through a clever network structure design and optimization strategies. When [11] was first proposed, it introduced new ideas that greatly improved the detection precision of small objects compared to previous algorithms. After improvements in the first generation, YOLOv2 [12] can detect up to 9000 classes. SSD [15] and YOLOv3 [13] divided their output into three feature maps for predicting objects of different sizes. Since then, YOLOv4 [14], YOLOv5 [1], YOLOv6 [16] and YOLOv7 [17] have made significant achievements in lightweight design, detection precision and comprehensive performance by integrating various advanced modules and strategies.

Many improved algorithms based on YOLOv5 have been proposed in various fields, which are capable of detecting different categories of small objects and achieving varying degrees of improvement. For example, Zhu [18] et al. proposed TPH-YOLOv5. A prediction head was appended and a transformer prediction head (TPH) with a self-focusing mechanism was supplied to increase the focus on dense areas. Compared to the previous SOTA method (DPNetv3), it achieved 1.81% improvement in the drone data set. Bai [19] et al. submitted two new object detection algorithms for traffic signs: YOLOv5-DH and YOLOv5-TDHSA. The former invented a decoupled head to speed up convergence, while the latter added a detection layer to improve detection precision. On the TT100k dataset, the F1 score improved by 0.04. Li [20] et al. created CME-YOLOv5 to improve the detection precision of fish. They used the fusion of coordinate attention mechanism and C3 convolution to pay more attention to object positioning information and improve the detection performance. Yang [21] et al. proposed the YOLOv5-CBS model for detecting river flotsam. The model created a new CCUB module that improves the adverse effects of light intensity and viewing angle on small object detection. Table 1 clearly shows the small object detection algorithms in different fields.

**Figure 1:**
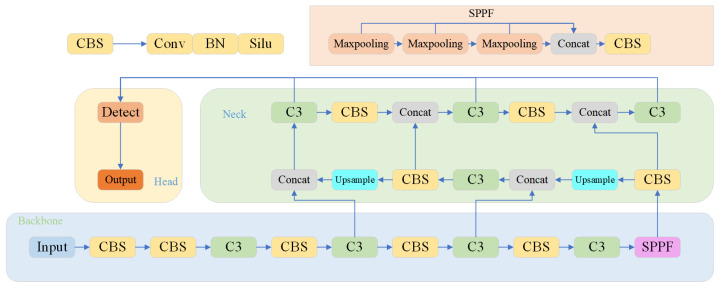
The original YOLOv5 network structure.

In this article, we proposed improvements to YOLOv5 in terms of both speed and precision. The YOLOv5 network comprises a backbone, neck and head for feature extraction, fusion, object detection, and classification, respectively. Figure 1 illustrates the original structure of the YOLOv5 network.

The enhanced MC-YOLOv5 algorithm incorporates three novel enhancements. Firstly, the CB module is integrated into the backbone network to replace the original C3 module and enhance detection accuracy. Secondly, the SNO module replaces a portion of FPN structure in the neck region, reducing missed object rates. Finally, the (A)DH structure substitutes for the original Detect function in the head section, accelerating training speed. Additionally, recent algorithms aimed at improving small object detection are summarized herein as a precursor to future research directions.

## 3. The Proposed MC-YOLOv5

YOLOv5 has emerged as one of the most widely adopted one-stage object detection algorithms following YOLOv3 [13], owing to its high stability, strong universality, and exceptional performance. However, there are still some challenges that need to be addressed. Firstly, small object detection suffers from a high rate of missed detections due to the loss of edge information caused by deep convolution and 32-fold down-sampling in two-dimensional images with smaller proportions of pixels for smaller objects than large ones. To address this issue, we proposed a novel CB feature extraction structure that preserves as much edge information for small objects as possible without significantly increasing parameters or floating-point arithmetic operations. This improvement enhances the precision of small object detection. In traditional detection methods, semantic information becomes clearer with an increase in convolutional layers for larger-sized objects; however, exceeding a certain number of convolutions leads to oversaturation. Therefore, the 32-fold down-sampling layer serves as the clearest conceptual layer for large-size objects while shallow convolutional layers provide clearer goal concepts for small objects. We introduced the SNO shallow network fusion method to further enhance the detection performance of small objects. The object detection results not only indicate confidence levels but also use anchor boxes to show object locations in the head section during detection tasks. Original YOLOv5 performed classification and localization tasks simultaneously which shortened training inference time but reduced classification and localization accuracy; however, the anchor-based Decoupled Head can significantly reduce error rates while improving precision in detecting small objects.

### 3.1. New CB Module

**Figure 2:**
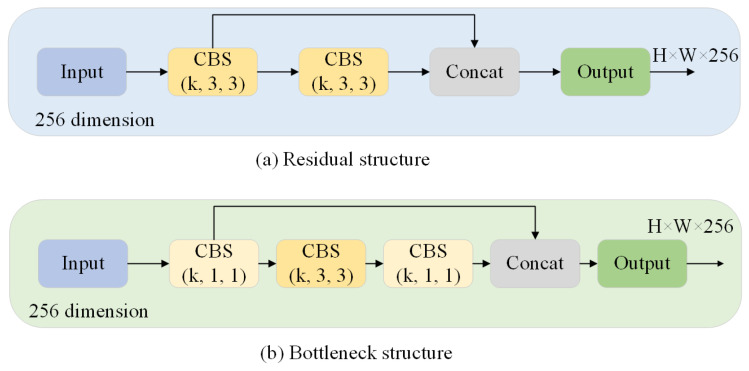
(**a**) The residual structure has 256 input channels and consists of two 3 × 3 convolution layers containing over a million parameters. (**b**) The bottleneck structure composed of two 1 × 1 convolutional layers sandwicted between a 3 × 3 convolutional layer. A 1 × 1 convolution realized the function of first dimension reduction and then dimension increased; thus, the 3 × 3 convolutional layer became the bottleneck with smaller input/output dimension. It has nearly 70,000 parameters. The number of the former was 17 times less than the latter.

Resnet [22] addressed the issue of gradient explosion or vanishing gradients that occur during multiple convolutions by incorporating residual elements through a shortcut mechanism to integrate the initial feature layer, thereby facilitating more effective feature extraction. In comparison to the building block structure employed in Resnet [22], the bottleneck structure significantly reduces computational complexity. An illustrative diagram depicting both the residual and bottleneck structures is presented in Figure 2.

**Figure 3:**
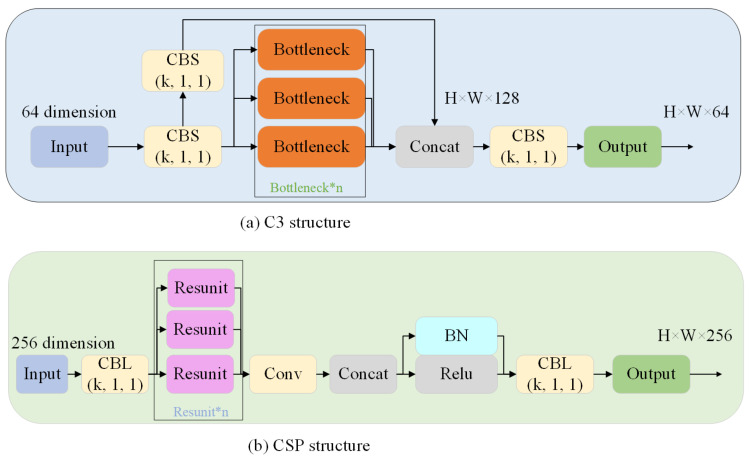
(**a**) The structure of C3, whose input is H × W × C. The C3 module contained three basic convolution layers and n bottleneck modules (n is determined by the co-configuration file and its network depth), and the activation function of basic convolution changed from LeakyRelu to Silu. (**b**) The structure of bottleneck-CSP, whose input is H × W × C. It consists of ordinary convolution and resunit structures.

In version 4.0 of YOLOv5, the bottleneck structure proposed in Resnet [22] was adopted along with C3. By taking the original YOLOv5s configuration file as an example, it was found that the C3 module reduced model reference by approximately 5.7% compared to Bottleneck-CSP while maintaining precision levels in full-size object detection. However, there has been no improvement in the feature extraction ability for small objects, and detection precision remains at a low level. A comparison between the C3 and bottleneck-CSP is illustrated in Figure 3.

**Figure 4:**
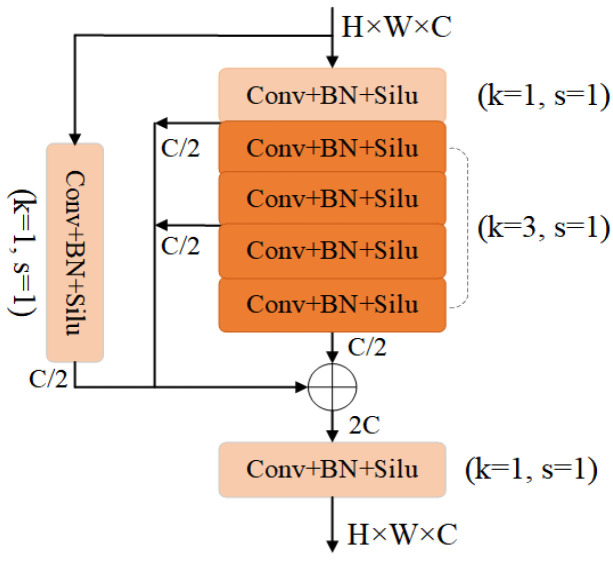
The structure of ELAN, whose input is H × W × C. It consists of six CBS. The srides of these CBS are either 1 or 3. The output size remains the same.

By enhancing the gradient propagation efficiency of the deep network to enhance its feature extraction capability, the Efficient Aggregation Layer Module (ELAN) was proposed in September 2022. The ELAN module [17] effectively mitigates the issue of model convergence caused by scaling and exhibits a more stable ability for model learning. However, due to its lengthy gradient update path, it introduces complexity to the network structure. Additionally, the substantial number of parameters hinders inference speed and convenience. Figure 4 illustrates the architecture of the ELAN module.

**Figure 5:**
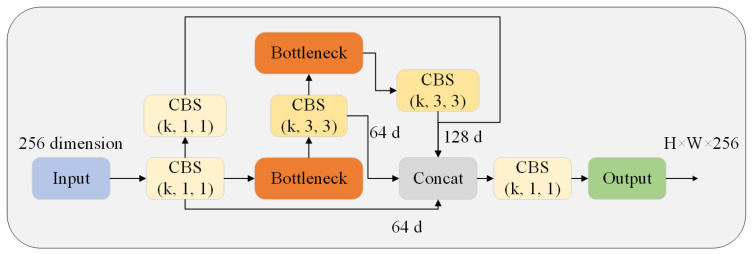
The structure of CB. It composed of some CBS modules with a convolutional kernel of 1 or 3 and 2 bottlenecks. The output size is consistent with the input size.

Based on existing research ideas, we retained the lightweight structure of the model while improving detection performance for small objects. However, detecting objects with small pixel proportions or in crowded areas remains challenging. To address this issue, we introduced a new CB structure based on C3 that applies multiple CBS structures to enhance feature extraction for edge information. We also incorporated a residual structure to prevent gradient anomalies and use bottleneck modules to reduce parameter numbers. This CB structure strikes a balance between training efficiency and detection precision (as shown in Figure 5), which we refer to as +CB in our experimental section.

### 3.2. The Revised Shallow Network Optimization Strategy (SNO)

**Figure 6:**
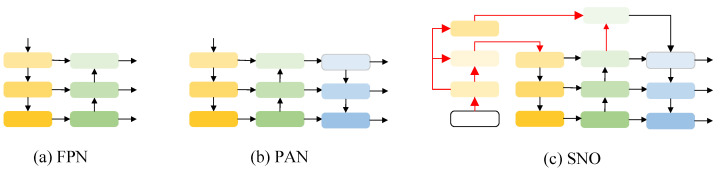
Schematic of the network fusion of FPN, PAN and SNO (ours).

The comparison of FPN, PAN and SNO structures is illustrated in Figure 6. YOLO feeds the RGB image into the backbone layer (yellow square) for multiple down-sampling and convolution operations. After completing an up-sampling process, it becomes FPN which was applied to earlier versions. Multiple fusion has been found beneficial to improve precision leading to the invention of PAN; however, excessive fusion results in overfitting. To solve the small object problem, we decideded to change the receptive field by taking input images with a size of 640 × 640 as an example. After 8× down-sampling, the image size became 80 × 80 pixels; similarly, after 16× down-sampling, it became 40 × 40 pixels with a receptive field of 16 × 16 pixels. Finally, after 32× down-sampling, it was reduced to an image size of 20 × 20 pixels with a receptive field of 32 × 32 pixels before going through FPN or PAN. As the receptive field increases, large objects are more easily detected; however, this is disadvantageous for mini and crowded small objects in datasets where redundant information leads to inaccurate anchor frame positioning and increased classification difficulty.

The SNO structure, as depicted in Figure 6c, undergoes an 8× down-sampling process resulting in a reverse reduction to 160 × 160. This layer is then combined with the 4× down-sampled feature layer and transmitted to the FPN network via CBS, necessitating an additional up-sampling operation by FPN. Ultimately, the PAN network inputs three layers of sizes 160 × 160, 80 × 80, and 40 × 40, respectively, for detecting ultra-small, small and medium-sized objects. In our experiments section, we refer to this architecture as +SNO.

### 3.3. The Decoupled Head Based on Anchor

**Figure 7:**
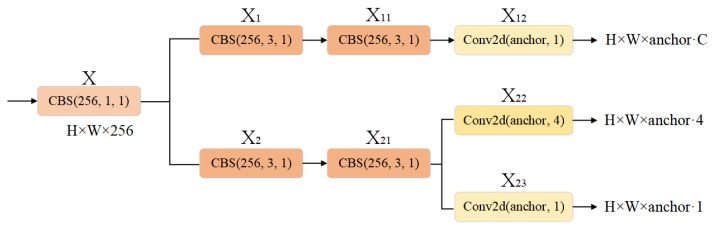
The structure of Decoupled Head based on anchor.

In the original YOLO framework, localization and classification tasks are performed simultaneously; however, this simultaneous execution often leads to spatial misalignment due to the inconsistent focus of these two tasks. Specifically, the classification task primarily emphasizes identifying the most similar category among extracted features, while localization focuses on refining boundary box parameters by prioritizing accurate position coordinates. When using the same feature map for both tasks, suboptimal results may be obtained. To address this issue and enhance convergence rate as well as precision, YOLOX [23] introduced a Decoupled Head structure as a replacement for the original Head component. Building upon previous insights, MC-YOLOv5 incorporates a Decoupled Head based on an anchor method which is illustrated in Figure 7.

**Figure 8:**
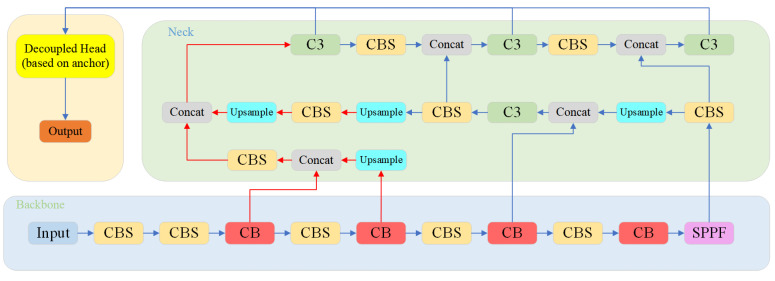
Structure of MC-YOLO network.

In general, MC-YOLOv5 has used the above three improvement points, and its network structure is shown in Figure 8.

## 4. Experiments

The experimental equipment utilized for this study consisted of a Windows 11 Professional Workstation version, with 16GB physical memory and 84GB virtual memory, an I5-12400F CPU, an NVIDIA GeForce RTX 3060Ti GPU with 8G of memory. All experiments were conducted using YOLOv5 6.1 based on PyTorch (1.10.0 + CU113), and the GPU was employed to accelerate training, validation and testing processes.

In comparison, we employed F1 score, mAP@0.5, and mAP@0.5:0.95 as evaluation metrics for assessing the algorithm’s detection accuracy, while FLOPs and inference time were utilized to evaluate the algorithm’s lightweight efficiency.

### 4.1. Datasets

To demonstrate the efficacy of our MC-YOLOv5 algorithm in detecting multi-class small objects, we utilized three publicly available datasets to train and validate its feasibility. Following the mainstream perspective [24], we defined an object size ranging from 16 × 16 pixels to 42 × 42 pixels as small objects, while objects smaller than 16 × 16 pixels were considered mini objects. Given that the experimental verification employed an image input size of 640 × 640 pixels, we specifically selected objects with a side length less than 640 × 0.065 pixels for evaluating the performance of MC-YOLOv5.

**Figure 9:**
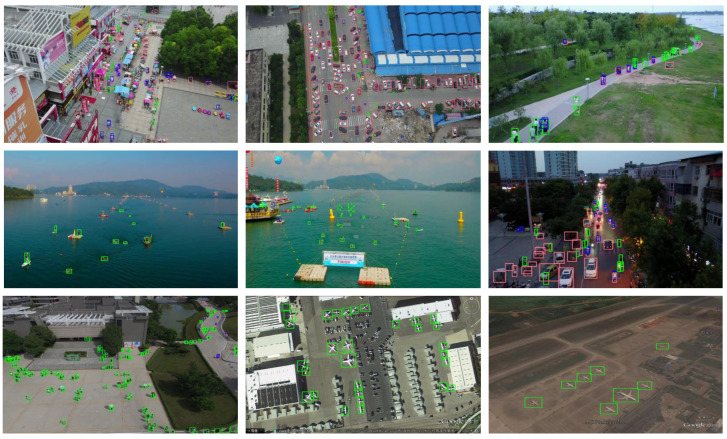
Some examples of object detection using MC-YOLOv5. Including and not limited to day, night, pedestrians, vehicles, water pedestrians, aircraft, etc. The objects in the figure are labeled with green or red anchor boxes.

**Figure 10:**
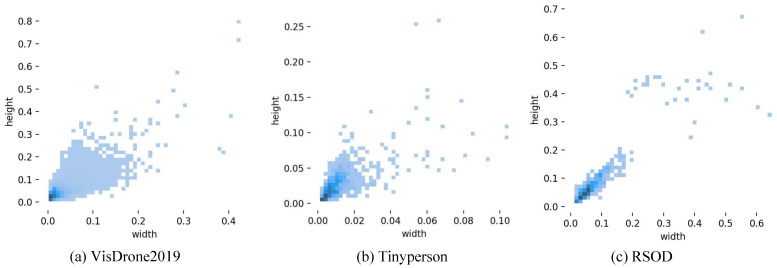
The plot illustrates the distribution of object sizes (width and height) in the dataset, where each size is represented by a blue square. Some areas are darker due to overlapping objects of the same size.

The VisDrone2019 dataset contains ten types of objects, including pedestrian, people, bicycle, car, van, truck, tricycle, awning-tricycle, bus, and motor. It includes not only daytime scenes, but also complex environments such as night scenes, and many cases where objects are too crowded to cover each other. Remove object pixel dimensions greater than 42 × 42 pixels, leaving only images that meet the criteria. We finally selected 6471 images for training and 548 images for verification and testing. TinyPerson is a publicly available small population data set focused on seaside pedestrian detection, with two categories of people at sea and people on land. The dataset is labeled with 1610 images. We selected 667 images for training and 50 images for verification and test. The RSOD dataset is a collection of remote sensing images taken from the air, which includes four types of objects: aircraft, playground, overpass, and oil drum. After reasonable screening, 842 images were selected for training and 94 images for verification and test. In this experiment, the dataset was partitioned into training, validation, and testing sets at a ratio of approximately 10:1:1. Figure 9 illustrates an example of partial detection, while Figure 10 displays the distribution map of object sizes across all three datasets.

### 4.2. Experimental Results and Comparison

According to the experimental setup, we evaluated the algorithm using mAP@0.5, mAP@0.5:0.95, Parameters (M), FLOPs (G), Times (ms) and F1 score. In addition to comparing MC-YOLOv5 with YOLOv5s and YOLOv5L, we also compared them with other mainstream algorithms such as YOLOv3 [13], YOLOv4 [14], YOLOv7 [17] and YOLOv8 on the VisDrone2019 dataset with maximum diversity at an image input size of 640 × 640 pixels. The original versions of YOLOv5s and YOLOv5L were used as benchmarks for training on the Visdrone data set without using official preset weights.

The YOLOv3 [13] algorithm is an earlier and more mature algorithm that was widely adopted by the YOLO framework, which introduces multi-scale detection heads for the first time. YOLOv4 [14], proposed by AlexeyAB’s team, incorporates enhancements such as Mosaic augmentation and other improvements in the input pipeline. On the other hand, YOLOv7 [17], a novel algorithm put forward by Wang et al., achieves significant accuracy improvement through efficient aggregation network utilization. While YOLOv3 [13] is not considered a lightweight model, it outperforms YOLOv4 [14] in terms of accuracy but still falls short compared to our MC-YOLOv5 (All).

In the realm of lightweight methods, MC-YOLOv5 (+CB) outperforms the original YOLOv5s in terms of mAP@0.5 and mAP@0.5:0.95 by 1.7% and 1.1%, respectively, while also boasting a more streamlined architecture with 8.5% fewer parameters and 3.1% fewer flops, resulting in a reduction of inference time by 1ms per image and an improvement in F1 score by 2%. It is worth noting that YOLOv4 [14] was included for comparison purposes despite its lack of lightweight design features, leading to lower accuracy and slower speed compared to other models as detailed in Table 2, which presents relevant indicators such as Parameters and Flops. An evaluation of the performance of MC-YOLOv5 and three other YOLO algorithms across three datasets is in Table 3.

Naturally, our objectives extended beyond lightweight enhancements. We placed greater emphasis on refining model accuracy, encompassing metrics such as mAP@0.5, mAP@0.5:0.95, and F1 score. MC-YOLOv5 (All), an amalgamation of three key improvements, exhibits significant advancements across all dimensions when compared to the original YOLOv5L framework. Notably, there is an 8.2% increase in mAP@0.5, a 5.3% increase in mAP@0.5:0.95, and a 7% increase in F1 score achieved by MC-YOLOv5 (All). Simultaneously, this method achieves a reduction in complexity with parameters reduced by 17%, flops reduced by 35%, and inference time decreased by 1.8 ms.

The results in Table 3 demonstrate that MC-YOLOv5 (All) achieves higher mAP@0.5 and mAP@0.5:0.95 on the Tinyperson dataset than the original YOLOv5L, with improvements of 1.2% and 1.07%, respectively. Due to the single background color and relatively small proportion of object pixels in the Tinyperson dataset, which has smaller dimensions compared to other datasets, the ELAN structure in the YOLOv7 model is susceptible to overfitting, resulting in decreased accuracy; however, MC-YOLOv5 effectively addresses this issue.

**Figure 11:**
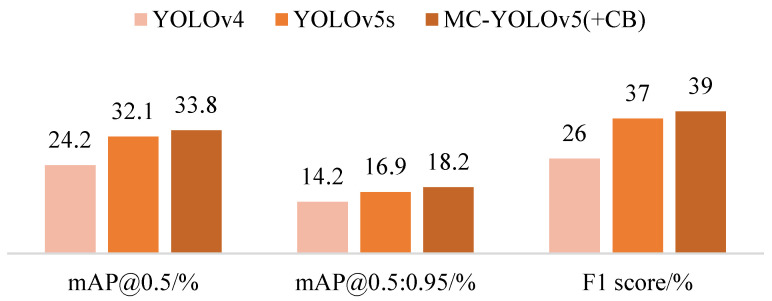
Lightweight performance comparison.

**Figure 12:**
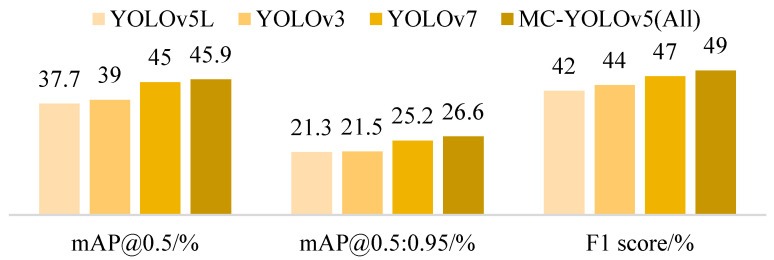
Standard level performance comparison.

**Figure 13:**
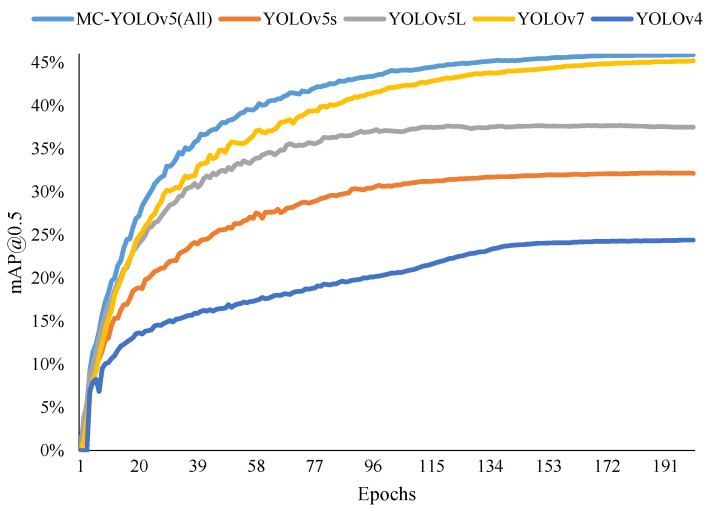
Some experimental results, including the comparison of the mAP@0.5 of the MC-YOLOv5 (All), YOLOv5s, YOLOv7 and YOLOv4 of these algorithms.

**Figure 14:**
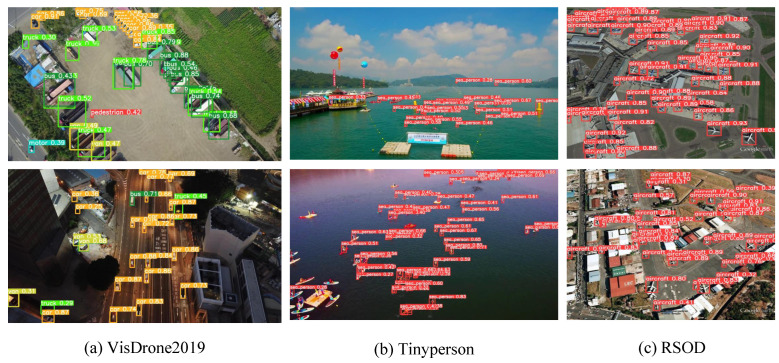
The detection performance of MC-YOLOv5 on three datasets is presented, with different categories represented by distinct colors in each group: (**a**) Car in orange, pedestrain in red, bus in dark green, truck in light green, motorbike in cyan, and van in light yellow; (**b**) sea_people depicted as red; and (**c**) aircraft also shown as red.

In order to present the advantages of our algorithm in a more intuitive manner, we visually compared the lightweight and standard algorithms using two-dimensional bar charts. The comparative plots are depicted in Figure 11 and Figure 12. Additionally, Figure 13 illustrates the mAP@0.5 value curves for various related algorithms during the training process on the VisDrone dataset. Furthermore, Figure 14 showcases the detection performance of MC-YOLOv5 on three different datasets.

### 4.3. Ablation Experiments

The ablation experiments were conducted in five groups, wherein the original YOLOv5 was modified into YOLOv5n, YOLOv5s, YOLOv5m, and YOLOv5L with varying network depth and width configurations. Gradually increasing the network depth and parameter count resulted in improved accuracy for each variant. In this study, we compared MC-YOLO with both YOLOv5s and YOLOv5L to strike a balance between accuracy and speed. Specifically, we used the original YOLOv5s as a reference for +CB analysis while employing the original YOLOv5L as a benchmark for +CB+SNO and All evaluations.

The mAP@0.5 values for all categories across the three datasets are presented in Table 4, with YOLOv5s and YOLOv5L serving as baselines. The floating value, indicated within parentheses, represents the relative improvement compared to the preceding column. Additionally, the average rank of the five models is displayed in the final row. Notably, based on this average rank analysis, it is evident that +CB structure outperforms YOLOv5s by more than one rank. Furthermore, both +CB+SNO and All achieve a higher ranking than YOLOv5L by more than one rank as well. These results provide compelling evidence for the significant advantages offered by our proposed improvements.

Compared to the other two datasets, the RSOD dataset exhibits a larger object size and less prominent edge information. Fortunately, our proposed CB structure proves beneficial for extracting object features, regardless of whether they are mini objects or medium and small objects. Furthermore, the decoupled head based on anchor enhances detection accuracy by improving both object localization and classification precision while being minimally influenced by object size.

**Figure 15:**
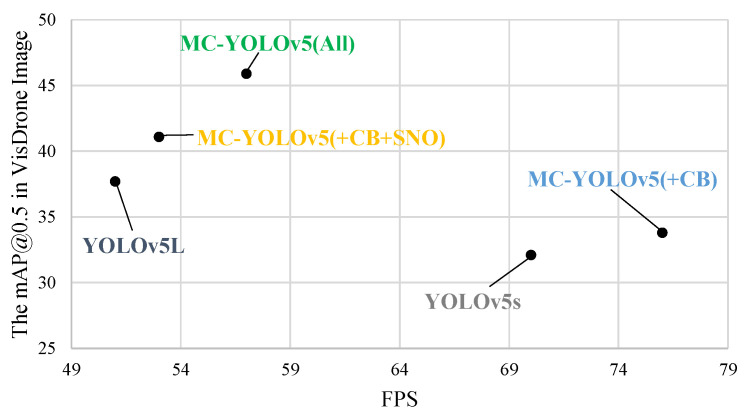
Comprehensive comparison.

Figure 15 shows the performance of the five methods in terms of real-time detection speed (FPS) and accuracy.

### 4.4. Discussion on Efficiency of MC-YOLOv5

The proposed algorithm, based on the YOLOv5 model, demonstrates superior performance in detecting multi-category small objects. This assertion is substantiated through rigorous contrast and ablation experiments conducted to evaluate and compare the efficacy of our models (MC-YOLOv5 (+CB), MC-YOLOv5 (+CB+SNO), and MC-YOLOv5 (All)) against state-of-the-art counterparts such as YOLOv5s and YOLOv5L. Our evaluation encompasses three diverse datasets: VisDrone2019, Tinyperson, and RSOD. Notably, the experimental results unequivocally establish that our three proposed enhanced models outperform the baseline models in terms of mAP@0.5 scores. To determine the statistical significance of MC-YOLOv5 compared to state-of-the-art models, we employed the nonparametric Friedman test [25,26] and corresponding post hoc Bonferroni–Dunn test [27,28], commonly used for comparing classifiers across multiple datasets.

The performance of the models employed in the ablation experiments was evaluated on three datasets using the Friedman test [25,26]. To determine the ranking of each algorithm on every dataset, algorithms were ranked from best to worst. In cases where multiple algorithms exhibited identical performance on a particular dataset, equal rankings were assigned.

The Friedman statistic can be calculated as follows:

(1) $$\chi_{F}^{2}=\frac{12N}{k(k+1)}\left[\sum_{j=1}^{k}r_{j}^{2}-\frac{k{\left(k+1\right)}^{2}}{4}\right]$$

where *N* and *k* are the number of datasets and algorithms, respectively. If there is no significant difference in the performance of the five algorithms, the aforementioned statistic follows a chi-square distribution with 3 degrees of freedom. However, upon calculating this statistic, it was found that its *p*-value is 0.029, which falls below the significance level of 0.05. Therefore, it can be concluded that there exists a significant difference in the performance of individual algorithms. It is evident that differences exist among all five algorithms and thus a post hoc test is necessary to determine statistical differences between their performances.

**Figure 16:**
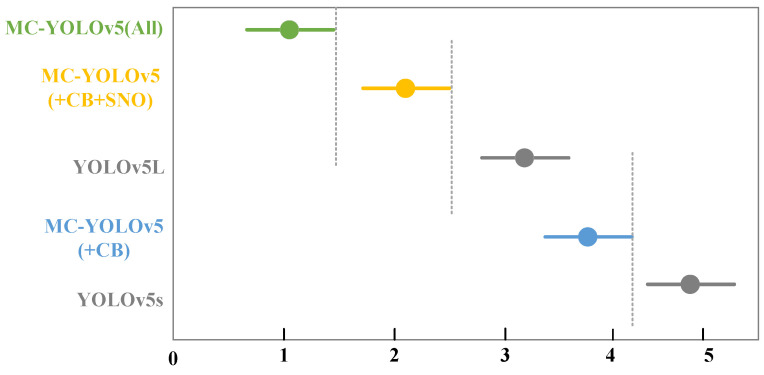
The average rank of the 5 methods.

The mean difference in rankings between MC-YOLOv5 (All) and +CB+SNO is 1.09, while the average ranking disparity between +CB+SNO and YOLOv5L is 1.07. Additionally, the average discrepancy in rankings between +CB and YOLOv5s is 1.12. Figure 16 illustrates the average ranks of these five methods.

**Figure 17:**
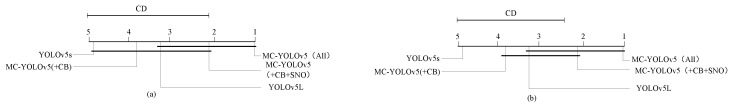
Where 1-5 represents the ranking of the algorithms. Critical difference (CD) comparison of MC-YOLOv5 (the control model) against other compared models with the Bonferroni–Dunn test, based on (**a**) mAP@0.5 with confidence level α = 0.05, CD = 2.896; (**b**) mAP@0.5 with confidence level α = 0.10, CD = 2.532 (any two models not connected by a thick black horizontal line are considered to have significant performance differences between each other).

Subsequently, a post hoc Bonferroni–Dunn test [27,28] was conducted to compare the models solely with the control models rather than among themselves. The Bonferroni–Dunn test evaluates the difference between each algorithm’s average ranking and a critical difference (CD). If this difference exceeds the domain value, it indicates that an algorithm with a higher average ranking is statistically superior to one with a lower average ranking and vice versa. Critical Difference is calculated as follows:

(2) $$CD=q_{\alpha }\sqrt{\frac{k(k+1)}{6N}}$$

where $q_{\alpha }$ denotes the critical value for $\frac{\alpha }{k-1}$. when *k* = 3, $q_{\alpha }$ = 2.241 for α = 0.05, and $q_{\alpha }$ = 1.960 for α = 0.10 [29]. The corresponding CD values, calculated according to Equation (2), are 2.896 and 2.532 for the proposed MC-YOLOv5 model and YOLOv5s, respectively. Figure 17 clearly demonstrates that the performance of the proposed MC-YOLOv5 model surpasses that of YOLOv5s on both evaluation metrics at both confidence levels.

## 5. Conclusions

Based on the YOLOv5 model, we proposed MC-YOLOv5, a multi-class small object detection algorithm that incorporates three innovations. Firstly, we introduced the new CB structure to replace the original C3 structure in the feature extraction stage, resulting in a more lightweight network model and improved detection speed. The SNO strategy optimizes feature fusion and addresses missed or false detections caused by dense distribution of small objects, thereby enhancing detection accuracy. Additionally, our decoupled head based on anchor separates classification and localization tasks into distinct steps, effectively considering both object position information and class information for improved model performance. In summary, compared to YOLOv5s, which prioritizes inference speed while maintaining basic accuracy, our +CB model focuses on achieving faster inference without compromising accuracy. Furthermore, MC-YOLOv5 (All) combines all innovations and exhibits significant improvements in both accuracy and speed compared to YOLOv5L. Moving forward, we aimed to further optimize this model for enhanced lightweightness while preserving its original accuracy. 

## Figures and Tables

**Table 1:** Comparison of several small object detection algorithms based on YOLOv5.

Models	Improvement	Classes
TPH-YOLOv5	+TPH	VisDrone2021(UAV)
YOLOv5-TDHSA	+T, +DH, +SA	TT100k& CCTSDB (traffic)
CME-YOLOv5	+CA, +EIoU	River floating garbage (private)
YOLOv5_CBS	+CCUB, +BiFPN, +SIoU	Fish (private)
MC-YOLOv5 (Ours)	+CB, +SNO, +(A)DH	VisDrone2019(UAV), Tinyperson(person), RSOD(airplane)

**Table 2:** Comparison of experimental results.

Methods	mAP@0.5	mAP@0.5:0.95	Parameters (M)	Flops (G)	Times (ms)	F1 Score
YOLOv4	24.2	14.2	64.3	143.2	58.6	0.26
YOLOv5s	32.1	16.9	7.0	15.8	14.1	0.37
**MC-YOLOv5 (+CB)**	**33.8**	**18.2**	**6.4**	**15.3**	**13.1**	**0.39**
YOLOv3	39.0	21.5	61.5	154.7	47.9	0.44
YOLOv5L	37.7	21.3	46.1	107.8	19.3	0.42
YOLOv7	45	25.2	36.5	103.3	19.6	0.47
**MC-YOLOv5 (All)**	**45.9**	**26.6**	**38.2**	**69.7**	**17.5**	**0.49**

**Table 3:** Accuracy of MC-YOLOv5 and three other YOLO algorithms on three data sets.

Datasets	Metrics	YOLOv5s	YOLOv3	YOLOv4	YOLOv5L	YOLOv7	MC-YOLOv5 (All)
Tinyperson	mAP@0.5	11.3	20.3	12.63	19.1	6.91	**20.3**
	mAP@0.5:0.95	2.6	5.22	3.64	4.8	1.52	**5.87**
RSOD	mAP@0.5	92.9	94.2	92.4	94.8	95.5	**96.7**
	mAP@0.5:0.95	61.6	66.8	59.3	66.6	63.8	**66.9**

**Table 4:** The mAP@0.5 of each method for all classes in three datasets.

Classes (Complete)	YOLOv5s (Baseline)	MC-YOLOv5 (+CB)	YOLOv5L (Baseline)	MC-YOLOv5 (+CB+SNO)	MC-YOLOv5 (All)
Pedestrian	40.3	40.9(+0.6)	46.5	50.5(+4)	**55.3(+4.8)**
People	32.1	33.5(+1.4)	36.6	38.2(+1.6)	**45.1(+6.9)**
Bicycle	9.9	10.7(+0.8)	14.4	17.6(+3.2)	**22.6(+5.0)**
Car	72.7	74.2(+1.5)	77	82.2(+5.2)	**84.1(+1.9)**
Van	33.4	37.2(+3.8)	41.4	44.7(+3.3)	**48.1(+3.4)**
Trunk	26.4	27.9(+1.5)	33.1	34.3(+1.2)	**39.3(+5)**
Tricycle	18.5	18.7(+0.2)	24.2	28.1(+3.9)	**32.2(+4.1)**
Awnin-tricycle	11.6	12.4(+0.8)	11.4	14.1(+2.7)	**18.3(+4.2)**
Bus	39.0	43.4(+4.4)	48.9	53.8(+4.9)	**60.4(+6.6)**
Motor	38.1	38.9(+0.8)	43.5	47.7(+4.2)	**54.0(+6.3)**
Sea-person	12.4	14.7(+2.3)	17.6	12.9(−4.7)	16.3(+3.4)
Earth-person	10.2	16.5(+6.3)	20.6	22.6(+2)	**24.4(+1.8)**
Aircraf t	94.8	95.4(+1.6)	95.2	95.5(+0.3)	**95.7(+0.5)**
Oil-tank	99.1	99.3(+0.2)	99.4	99.4(−)	**99.4(−)**
Overpass	78.3	89.3(+11)	87.1	92.3(+5.2)	**94.9(+2.6)**
Playground	99.5	99.5(−)	97.5	99.6(+2.1)	**99.7(+0.1)**
Average Rank	4.84	3.72	3.22	2.15	**1.06**

## Data Availability

Not applicable.
